# Overweight and Obesity among Women by Economic Stratum in Urban India

**Published:** 2014-03

**Authors:** Jitendra Gouda, Ranjan Kumar Prusty

**Affiliations:** International Institute for Population Sciences, Mumbai, India

**Keywords:** Body mass index, Economic status, Urban women, India

## Abstract

Using data of the third round of the National Family Health Survey (NFHS) 2005-2006, this study examined the prevalence of overweight and obesity among women from different economic strata in urban India. The study used a separate wealth index for urban India constructed using principal components analysis (PCA). The result shows that prevalence of overweight and obesity is very high in urban areas, more noticeably among the non-poor households. Furthermore, overweight and obesity increase with age, education, and parity of women. The results of multinomial logistic regression show that non-poor women are about 2 and 3 times more at risk of being overweight and obese respectively. Marital status and media exposure are the other covariates associated positively with overweight and obesity. Thus, the growing demand which now appears before the Government or urban health planners is to address this rising urban epidemic with equal importance as given to other issues in the past.

## INTRODUCTION

Obesity and overweight have become a global epidemic now. According to the World Health Organization (WHO), there will be about 2.3 billion overweight people aged 15 years and above and over 700 million obese people worldwide in 2015. Overweight and obesity are the fifth leading risk of deaths, resulting in around 2.8 million deaths of adults globally every year. In addition, 44% of the diabetes burden, 23% of the ischaemic heart disease, and between 7% and 41% of certain cancer burdens are attributable to overweight or obesity (1). The causes and co-morbidities of overweight or obesity are rampant and have many commonalities among populations. Although identifying firm causes of this epidemic is a difficult task, the most obvious factors leading to overweight or obesity are excessive intake of energy-dense food, sedentary lifestyle, and lack of physical activity (2,3).

The problem of overweight or obesity is no more restricted only to the developed world. Presently, the epidemic poses new challenges in developing countries and urges immediate attention and prevention. These countries face double burden of nutritional problems as they are yet to solve the erstwhile problems of undernutrition and hunger (4). Many scholars explained it in the perspective of the “nutritional transition in developing countries, or the shift from traditional diets and lifestyles to Western diets” (i.e. highly-saturated fats, sugar, and refined foods) and the combination of reduced levels of physical activity, transport facilities, better healthcare, and increased stress, particularly in the rapidly-growing urban populations (5-7). Furthermore, a significant positive correlation has been observed between better economic status and composition of diet consumed. People from economically better-off families are more likely to adopt sedentary lifestyle and intake energy-dense food (7-13). For example, in China, along with its rapid urbanization, the average intake of energy-dense food has increased over the last decade in urban population. In addition, reduced physical activity at work due to mechanization, improved motorized transport, and preferences of viewing television for longer duration have resulted in positive energy balance in people of most of the Asian countries (10-13).

As in most developing nations, India is struggling to eradicate the problem of undernutrition and anaemia. Meanwhile, the country already witnessed the overweight and obesity problem. India has more than 30 million obese people, and the number is increasing alarmingly (14-16). The problem is more acute among women than men. In urban India, more than 23% of women are either overweight or obese, which is higher than the prevalence among men (20%) (16). Thus, the country is burdened with two different nutrition-related health problems (4). It has to grapple with the problem of undernutrition and anaemia in one hand and overweight or obesity on the other (17-19). Unlike the developed countries where obesity is generally concentrated among the low/middle-income groups, elevated adiposity levels in developing countries are more associated with women from the richer sections of the society, noticeably in urban areas (2,19-21).

However, evidence also suggests that unplanned urbanization in developing countries, like India, leads a large proportion of people to live below the poverty line. Moreover, they live in those deficient areas which have limited availability of or accessibility to basic civic amenities (22). Further, they exhibit different disease and health patterns from their counterparts living above the poverty line or in better-off areas (23,24). India has more than 30% of the urban population, which is projected to increase to 900 million or 55% by 2050 (25,26). Due to rapid and unplanned urbanization, intra-urban socioeconomic disparities are rising, and health inequality among urban dwellers is emerging as a new challenge (27). Hence, any insightful assessment on defining section of the population with high prevalence of overweight and obesity in the urban setting in India will be helpful for the urban health planners to tackle the problem. Therefore, the present study attempts to shed light on overweight and obesity among women in urban India, with special reference to their economic strata.

Objectives of the study are to understand the sociodemographic differentials of overweight and obesity among women in urban India and selected cities by their economic stratum and to find out different covariates associated with overweight and obesity among urban women in India.

## MATERIALS AND METHODS

The study used data of the third round of the National Family Health Survey (NFHS) 2005-2006 for the assessment of overweight and obesity among women in urban India. The survey is the Indian version of Demographic Health Survey (DHS) which is conducted in more than 80 countries all-over the world. NFHS-3 collects information from a nationally-representative sample of 109,041 households—124,385 women of reproductive age (15-49 years). The sample is a multistage cluster sample with an overall response rate of 98%. Details of sampling design, including sampling frame and sample implementations, are provided in the basic survey report for all India (16).

For the present study, ever-married women aged 15-49 years in urban areas were considered. All 50,639 valid cases, representing urban areas, were taken into account, and the missing values were ignored. The analysis is based on the economic stratum. Thus, estimating economic condition of ever-married women in urban India is a prior condition for this study. The direct measure of economic status of any household or individual is income or consumption expenditure, which is not available in the dataset used. So, an alternative measure is adopted by surveyors based on various economic proxies, such as household amenities, housing conditions, and consumer durables. All these proxy variables are used in a composite index and referred as the wealth index and are widely used in population and health analyses (23,28,29). However, many studies have documented the limitation of deriving such a single wealth index at the national level due to variation in the economic situation among population representing different geographic regions (30,31). Moreover, the economy in urban areas is more diverse than in the rural areas (32,33). Therefore, we constructed a new wealth index for urban India for this study.

### Urban poor and non-poor: cutoff points

To demarcate urban poor and non-poor, a set of consumer durables, household amenities, and housing qualities based on the theoretical importance and statistical significance were selected. The theoretical rationale refers to the sensitiveness of the variables to urban areas. After selecting the variables, principal components analysis (PCA) was used in estimating the wealth index. From the composite wealth index, a percentile distribution was obtained and used for demarcating the poor and non-poor in urban India (34).

The cutoff point to demarcate the poor and non-poor in urban India is equated with the official estimates of poverty (time periods coinciding with the surveys) derived from consumption expenditure data by the Planning Commission of the Government of India. Accordingly, 26% of the population in 2004-2005 (based on uniform recall period) is classified as urban poor in the third round of the NFHS (2005-2006) (34). In recent years, a number of studies have used the official estimates of poverty to demarcate the poor and non-poor in large-scale surveys (35,36).

### Outcome variables

*Overweight and obesity*: In NFHS-3, all ever-married women, aged 15-49 years, were weighed using a solar-powered scale with an accuracy of ±100 g. Their heights were measured using an adjustable wooden measuring board, specifically designed to provide accurate measurements (to the nearest 0.1 cm) in a developing-country field situation. The data on weight and height were used in calculating the body mass index (BMI). Women who were pregnant at the time of the survey or women who had given birth during the two months preceding the survey were excluded (15,16). BMI can be used in estimating the prevalence of underweight as well as the prevalence of overweight and obesity. As per the definition given by World Health Organization, a BMI of less than 18.5 kg/m^2^ is defined as underweight, indicating chronic energy deficiency. BMI in the range of 18.5 and 24.9 kg/m^2^ is defined as normal, 25.0 and 29.9 kg/m^2^ as overweight, and more than 30.0 kg/m^2^ as obese (37).

Based on these cutoffs, the present study used a three-category variable of nutritional status of women, merging underweight and normal to indicate ‘not obese’ while keeping all others the same as ‘overweight’ and ‘obese’.

### Predictor variables

The survey collects information on a number of demographic and socioeconomic factors, which could potentially affect the nutritional status of women. The variables which are included in this analysis are: age of respondent, religion, caste, educational attainment, marital status, parity, work status, region, and exposure to media. Listening to radio, reading newspapers, and watching TV are used in defining exposure to media in the study (17-18).

### Statistical analysis

Descriptive statistics are used for knowing the level and differentials of overweight and obesity among the poor and non-poor ever-married women by different sociodemographic characteristics. The results are presented in percentages. Multinomial logistic regression analysis is used in estimating the adjusted effects of selected socioeconomic and demographic covariates on the prevalence of overweight and obesity among the urban women in India. The multinomial regression was used due to the nature of the outcome variable. The outcome variable has three categories, namely not obese, overweight, and obese (coded as 0, 1, and 2 respectively). The results are presented in the form of relative risk ratio (RRR), with 95% of confidence interval. The relative risk (RR) explains the probability that a woman of an exposed group will be overweight or obese relative to the probability that a woman of an unexposed group will develop the same. In all our analyses, weights are used for restoring the representativeness of the sample. The analyses are done with the help of SPSS (version 20.0) and STATA (version 10) statistical packages.

## RESULTS

### Overweight and obesity among women in urban india

The prevalence of overweight and obesity is higher among urban women than their rural counterparts in India. More than 23% of women in the urban area are either overweight or obese compared to only 7% of women in rural areas (Figure). More than one-sixth of women in urban area are overweight, and around 6% of women are obese. The problem is more acute among the non-poor than the poor in urban India. For example, one-fifth of the women from non-poor households are overweight compared to less than one-tenth of the women from poor households. Moreover, 7% of non-poor and only 2% of poor women are obese in urban India (Table 1).

Among mega cities in India, Chennai has the highest (39%) proportion of overweight or obese urban women, followed by Hyderabad (34%), and Kolkata (30%). Furthermore, it is observed that non-poor women across all selected cities have higher prevalence of overweight and obesity than their counterparts from poor households (Table 1).

### Sociodemographic differential in overweight and obesity among poor and non-poor women in urban India

Comparing women of different age-groups across the economic strata of households, it is observed that women at later age (35+ years) are more overweight or obese than the reference group in 15-24 years. However, women from non-poor households at later age are more overweight or obese than their counterparts from poor households. The prevalence of overweight or obesity increases analogously with each additional age of women; yet, the increase is much higher for non-poor than the poor. Furthermore, non-poor women across all religions have higher proportion of overweight or obesity than their counterparts from poor households. Women from non-poor households, irrespective of their educational achievements, are more overweight or obese than their counterparts from poor households. However, women with higher education across the economic backgrounds have higher proportion of overweight or obesity than women with any other educational achievements.

**Figure.**
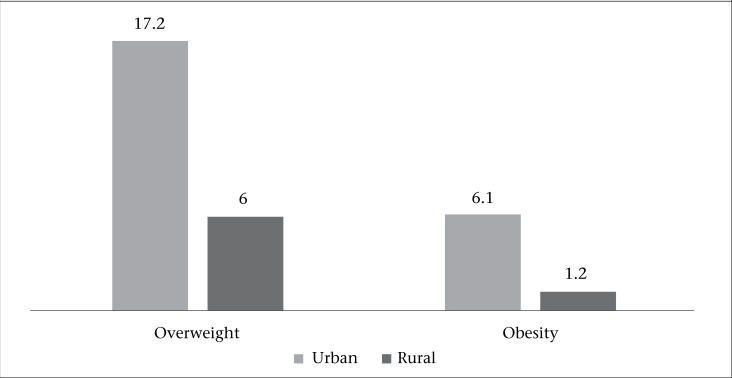
Rural-urban differential in the prevalence (in percentage) of overweight and obesity among women in India, 2005-2006

**Table 1.** Overweight and obesity (in percentage) among women in major cities in India by economic status, 2005-2006

BMI status	Economic status	Chennai	Kolkata	Hyderabad	Delhi	Mumbai	Urban India
	Poor	21.1	13.2	15.6	8.6	12.5	8.8
Overweight	Non-poor	29.2	25.2	25.0	20.5	20.4	20.0
	Total	26.8	23.0	23.0	19.2	19.0	17.3
	Poor	5.3	2.9	5.2	2.1	3.6	2.1
Obesity	Non-poor	15.1	7.7	11.8	8.7	8.8	7.1
	Total	12.2	6.8	10.4	8.0	7.9	5.9
Poor (n)		699	487	664	319	291	11516
Non-poor (n)		1,216	1,792	2,084	1,965	1,424	39,123
Total urban (N)		1,915	2,279	2,748	2,284	1,715	50,639

Women from non-poor households, irrespective of work status, are more overweight and obese than their counterparts from poor families. Yet, women who are not engaged in any income-generating activities across economic backgrounds are more likely to be overweight or obese than working women. The only exception is the non-poor and women not working (19.8%), who have a slightly lower proportion of overweight than working women (20.4%). Parity and risk of being overweight or obese among women is positively related as evident in the study. Furthermore, non-poor women across parities are more overweight or obese than their counterparts from poor households. Women with 3 and more children from non-poor households (26% and 11%) have higher prevalence of overweight and obesity respectively than women in the same parity from poor households (11% and 3%). Media exposure and risk of being overweight and obese are positively associated in India. Women with media exposure across all economic backgrounds have higher proportion of overweight and obesity than their counterparts without media exposure. However, non-poor women with media exposure (20% and 7%) are more overweight and obese than women from poor households with media exposure (9% and 2%). Non-poor women across all regions in India are more overweight and obese than their counterparts in the poor households. Moreover, women from southern region, irrespective of economic backgrounds, have higher prevalence of overweight and obesity than women from any other regions in India (Table 2).

### Multivariate analysis

The adjusted effect of selected demographic and socioeconomic covariates on the risk of being overweight and obese among women in India is presented in Table 3. Comparing poor and non-poor women, it is observed that non-poor women are relatively 2.18 times (p<0.01, CI 2.016-2.366) and 2.84 times (p<0.01, CI 2.449-3.302) more likely at risk of being overweight and obese respectively than their poor counterparts in urban India. The risk of being overweight (RRR=5.28, CI 4.787-5.816) and obese (RRR=12.31, CI 10.163-14.904) is more among women at later ages (35+ years) than the reference group in 15-24 years. Muslims (RRR=1.2, CI 1.086-1.275) and women from other religions (RRR=1.3, CI 1.148-1.462) are more likely to be overweight or obese than Hindu women. Furthermore, women from other (upper) castes groups are more overweight or obese than their counterpart SC/ST women in India. Comparing women by their educational achievements, it is evident that women with higher education have higher relative risk ratio (RRR=1.97 and 2.39) than the women with no education. Furthermore, married women are 1.86 and 2.14 times more likely to be overweight or obese respectively than the never-married women. Women with media exposure are 1.65 and 1.45 times more likely at risk of being overweight and obese than women without media exposure. Women from southern India are 1.41 times (p<0.01, CI 1.299-1.534) and 1.48 times (p<0.01, CI 1.301-1.672) more likely at risk of being overweight and obese than the reference women from the northern region.

## DISCUSSION

The primary objective of this study was to assess overweight and obesity among women in urban India, with special reference to their economic status. The study found that higher proportions of women in urban India are either overweight or obese than their counterparts from rural area. The problem is noticeably higher among affluent households than poor families. This generally contrasts with the findings of other studies conducted on the similar issues in Western and African countries where the poor are found to be more overweight or obese than the affluent (19,38). Nevertheless, a number of studies conducted in developing countries, especially in Asia, support the findings of this study, viz. the affluent are more overweight or obese than the poor (13,18) .

In the mega cities, the situation is alarming. Many women are either overweight or obese in the selected cities studied in India. This condition could well be compared with many other developed nations where the prevalence of overweight and obesity is accumulating steadily (39,40). The reasons behind non-poor women for being overweight or obese could be many in India. In a nutshell, rising income due to increasing participation in employment and improving socioeconomic status helps women to opt for sedentary lifestyle which is considered to cause weight gain (5,6,41,42).

Along with a number of studies, this study equally opined that fraction of overweight and obesity increases with age, education, and parity of the women (19,43,44). The multinomial analysis found that women aged 35 years and above are 5 times more likely to be overweight and 12 times more likely to be obese than women of 15-24 years. Many studies have attempted to determine the causes behind this association between overweight or obesity and demographic covariates. Among all, physical activity declines, along with metabolic rate, in the middle years of women. On the other hand, the energy requirement decreases; therefore, even regular or routine eating may lead to weight gain. In addition, the established cultural or social values with respect to care and diet given during and after pregnancy help women to gain more weight than ever. Furthermore, newly-married women at young age are more health-conscious and involved in more physical activity than women at older ages with children. This might be another important reason for weight gain after childbirth among women (3). The higher-educated women are two times more likely to be overweight or obese than women with no education (Table 3). Higher education opens better employment opportunities for women and leads to be self-dependent and for further improvement in socioeconomic status. This possibly helps women live a life which involves less physical activity and helps access energy-dense food which is considered to cause overweight or obesity (43,44). Women with higher parity are more overweight or obese in India. This generally implies that women's higher age with declining physical activity helps accumulate more weight.

**Table 2.** Percentage distribution of overweight and obesity among poor and non-poor women in urban India, 2005

Background characteristics	Overweight		Obesity		Sample-size (N)	
	Poor	Non-poor	Poor	Non-poor	Poor	Non-poor
Age of respondent (completed years)						
15-24	3.5	7.6	0.4	1.5	3,380	9,798
25-34	9.9	21.0	2.1	6.0	2,848	8,774
35+	14.2	31.5	4.2	13.7	2,727	9,714
Religion						
Hindu	8.5	20.4	2.0	7.0	6,369	20,144
Muslim	10.4	19.8	2.7	8.1	1,685	3,728
Christian	7.7	15.7	0.7	4.8	673	2,893
Others	6.5	23.0	3.7	9.6	214	1,490
Caste						
SCs^1^ & STs^2^	6.6	15.3	1.4	3.6	2,924	5,974
OBCs^3^	9.8	19.2	2.3	7.0	3,444	8,103
Others	9.4	22.2	2.4	8.6	2,224	13,057
Education						
No education	8.7	20.1	2.5	6.2	3,941	3,363
Up to primary	8.2	21.4	1.9	7.1	1,726	2,640
Up to secondary	9.0	18.5	1.6	7.1	3,128	16,098
Up to higher	11.9	23.1	1.9	7.4	159	6,182
Work status						
Not working	8.9	19.8	2.2	7.5	5,501	20,939
Working	8.6	20.4	1.8	5.8	3,448	7,293
Marital status						
Never married	4.0	7.5	0.3	1.6	1,940	8,230
Married	10.1	25.1	2.6	9.3	7,017	20,057
Parity						
0	5.3	9.5	0.7	2.3	2,570	9,993
1-2	9.8	25.5	2.3	9.0	2,635	10,568
3+	10.4	25.9	2.9	10.5	3,751	7,726
Media exposure						
No exposure	5.8	15.0	1.9	4.7	1,467	553
Have exposure	9.4	20.1	2.1	7.1	7,486	27,732
Region						
North	9.6	21.6	2.0	8.8	1,030	6,216
Northeast	6.5	15.5	1.0	2.9	1,255	4,451
East	7.0	20.0	0.8	6.0	1,241	2,600
West	7.7	18.8	3.0	7.1	1,112	6,078
Central	5.9	17.4	0.9	5.7	1,464	3,606
South	12.1	24.9	3.4	9.9	2,854	5,335

1 Scheduled castes;

2 Scheduled tribes;

3 Other backward classes

**Table 3.** Multinomial logistic regression showing relative risk of overweight and obesity among women in urban India, 2005-2006

Covariate	Overweight		Obesity	
	RRR	(95% CI)	RRR	(95% CI)
Economic background				
Poor®				
Non-poor	2.18***	2.016-2.366	2.84***	2.449-3.302
Age (completed years)				
15-24®				
25-34	2.59***	2.368-2.847	3.94***	3.260-4.755
35+	5.28***	4.787-5.816	12.31***	10.163-14.904
Religion				
Hindu®				
Muslim	1.18***	1.086-1.275	1.37***	1.214-1.552
Christian	0.86	0.774-0.963	0.86	0.713-1.041
Others	1.295**	1.148-1.462	1.71***	1.437-1.024
Caste				
SC^1^ & ST^2^®				
OBC^3^	1.08**	0.997-1.170	1.31***	1.135-1.502
Others	1.33***	1.237-1.438	1.73***	1.510-1.969
Education				
No education®				
Primary	1.18***	1.069-1.301	1.19**	1.004-1.400
Secondary	1.53***	1.409-1.658	2.03***	1.777-2.319
Higher	1.97***	1.783-2.173	2.39***	2.030-2.801
Marital status				
Never-married®				
Married	1.86***	1.636-2.112	2.14***	1.680-2.729
Parity				
0®				
1-2	1.02	0.918-1.143	0.95**	0.793-1.146
3+	0.97	0.866-1.096	0.99**	0.815-1.201
Work status				
Not working®				
Working	0.83***	0.784-0.882	0.65***	0.591-0.719
Media exposure				
No exposure®				
Have exposure	1.65***	1.417-1.918	1.45***	1.124-1.880
Region				
North®				
Northeast	0.77***	0.693-0.846	0.41***	0.339-0.491
East	0.86**	0.776-0.946	0.61***	0.518-0.720
West	0.85***	0.780-0.918	0.82	0.724-0.926
Central	0.82**	0.749-0.901	0.66***	0.571-0.769
South	1.41***	1.299-1.534	1.48***	1.301-1.672

® Reference group;

**p<0.05;

***p<0.01;

1 Scheduled castes;

2 Scheduled tribes;

3 Other backward classes; Pseudo R^2^=0.1291

Women with media exposure are about two times more at risk of being overweight or obese. This corroborates the findings of many other studies that proportion of overweight and obesity increases with media exposure (43). Media, in general, are a powerful tool which educates the mass on a number of important aspects, including healthy life practices. However, it also gives exposure to a number of energy-saving machineries, energy-dense or junk food items, which tend to influence women to adopt; this is considered a leading cause of overweight or obesity. Moreover, viewing television for longer duration can also increase the physical inactiveness and helps in weight gain. This is more common among the non-poor women since they have the ability to pay for all these expensive energy-dense food and other luxuries in urban India. Women in southern regions are more overweight and obese than women from other regions of India. Southern states in India have better socioeconomic indicators than other states. In these states, female education is comparatively higher than other states in India (45,46). Moreover, the proportion of women living below the poverty line is comparatively less in southern than the northern or eastern regions in India (47). So, having this favourable environment in these states, women apparently enjoy a better life or can have a sedentary lifestyle which further may lead to overweight or obesity (47).

In addition, this study can correctly conclude that married women are more overweight or obese. As an attempt to find the possible reasons behind this, a study concluded that exiting the dating market decreases one's incentive to maintain their appearance and leads to an increase in body-weight. However, the authors humbly appeal to the readers not to use the paper as opprobrium against marriage (48).

### Limitations

There are a number of measurement issues which need to be kept in mind while considering the findings of this study. First, the survey considered only the weight and height of women to measure the prevalence of overweight and obesity in India. However, there are many other sophisticated means to determine the overweight and obesity condition of a woman in a better way. Waist-circumference is one among those tools which can give a better measurement on these issues, especially in Asian region (49,50). Second, the survey collected limited information on lifestyle, physical activity, and diet. Although the demographic, socioeconomic and lifestyle factors incorporated in this study may capture much of the variation, more detailed information on these subjects in future studies can help understand the causes of overweight and obesity better.

### Conclusions

The study found that the problem of overweight and obesity is more of an urban concern. Another critical outcome of the study is that women of non-poor households are more overweight and obese than their counterparts from poor families. However, India's health policy often follows pro-poor and pro-rural approach and, thus, merely overlooks the problem of overweight and obesity. For an illustration, the flagship programme National Health Rural Mission (NRHM), funded by central government, has a number of building blocks or measures to address anaemia and undernutrition prevalent among women and children in rural India. Yet, the programme does not recognize the growing epidemic of overweight and obesity among women in urban India. With this backdrop, the growing demand which appears before the Government or the urban health planners is to address this rising epidemic with equal importance. A timely prevention will reduce the burden of many chronic co-morbidities, like diabetes, cardiovascular diseases, hypertension, and infertility on the health system in India (51). This can be achieved either through undertaking separate urban health programme or incorporating special clause in the proposed National Urban Health Program, citing the importance of healthy diet and physical exercise.

## ACKNOWLEDGEMENTS

The authors are grateful to Dr. Sanjay K. Mohanty and Mr. Abhishek Kumar for their constructive comments and suggestions on various sections of the paper. Authors would also like to thank the editors and two anonymous reviewers for their suggestions towards improvement of the paper.
